# The Emerging Role of Ferroptosis in Liver Diseases

**DOI:** 10.3389/fcell.2021.801365

**Published:** 2021-12-14

**Authors:** Si Chen, Jun-yao Zhu, Xin Zang, Yong-zhen Zhai

**Affiliations:** ^1^ Department of Neurology, The Fourth Affiliated Hospital of China Medical University, Shenyang, China; ^2^ Department of Infectious Disease, Shengjing Hospital of China Medical University, Shenyang, China

**Keywords:** ferroptosis, cell death, liver diseases, fibrosis, hepatocellular carcinoma

## Abstract

Ferroptosis is a newly discovered type of cell death mediated by iron-dependent lipid peroxide. The disturbance of iron metabolism, imbalance of the amino acid antioxidant system, and lipid peroxide accumulation are considered distinct fingerprints of ferroptosis. The dysregulation of ferroptosis has been intensively studied in recent years due to its participation in various diseases, including cancer, kidney injury, and neurodegenerative diseases. Notably, increasing evidence indicates that ferroptosis plays different roles in a wide spectrum of liver diseases. On the one hand, inhibiting ferroptosis may counteract the pathophysiological progression of several liver diseases, such as alcoholic liver injury, nonalcoholic steatosis hepatitis and fibrosis. On the other hand, inducing ferroptosis may restrict the emergence of secondary resistance to current medicines, such as sorafenib, for hepatocellular carcinoma (HCC) therapy. Here, we summarize the biological characteristics and regulatory signalling pathways of ferroptosis involved in liver disease. The current available medical agents targeting ferroptosis, including inducers or inhibitors applied in liver diseases, are also reviewed. This work aims to provide new insight into the emerging role of pathogenesis and therapeutic approaches for liver diseases.

## Introduction

In 2012, ferroptosis, a new type of cellular programmed death that differs from apoptosis and cellular autophagy, was proposed by Dr. Brent R. Stockwell’s team (Dixon et al., 2012). When intracellular free iron increases due to iron metabolic disorders, iron catalyses the production of reactive oxygen species (ROS) through the Fenton reaction, which further promotes lipid peroxidation, leading to lipid peroxide aggregation (Dixon et al., 2012; Stockwell et al., 2017; Jiang et al., 2021). Then, the depletion of glutathione (GSH) and the decrease in glutathione peroxidase 4 (GPX4) activity impede the metabolism of lipid peroxides via the glutathione reductase reaction catalysed by GPX4, leading to the destruction of the cell membrane integrity and eventually inducing ferroptosis (Dixon et al., 2012; Stockwell et al., 2017; Jiang et al., 2021). Morphologically, ferroptosis is characterized by increased mitochondrial membrane densities and vanished mitochondrial cristae without obvious changes in nuclear morphology (Cao and Dixon, 2016). The accumulation of ROS, depletion of GSH, inhibition of GPX4 activity, and increased release of polyunsaturated fatty acids (PUFAs) are regarded as the biochemical characteristics of ferroptosis (Liu J. et al., 2021; Tang D. et al., 2021; Wu et al., 2021).

In recent years, several mediators and signalling pathways involved in ferroptosis have been intensively identified (Yan et al., 2021). First, the role of redox homeostasis and lipid metabolism in ferroptosis has been highlighted. The cystine/glutamate antiporter system (system X_C_^−^) located in the cell membrane participates in the synthesis of GSH, and GPX4 uses GSH as a substrate to restore lipid peroxides to normal lipids (Cao and Dixon, 2016; Stockwell et al., 2020). Thus, the inhibition of either system X_C_^−^ or GPX4 could cause ROS and lipid peroxide aggregation and initiate ferroptosis.

**FIGURE 1:**
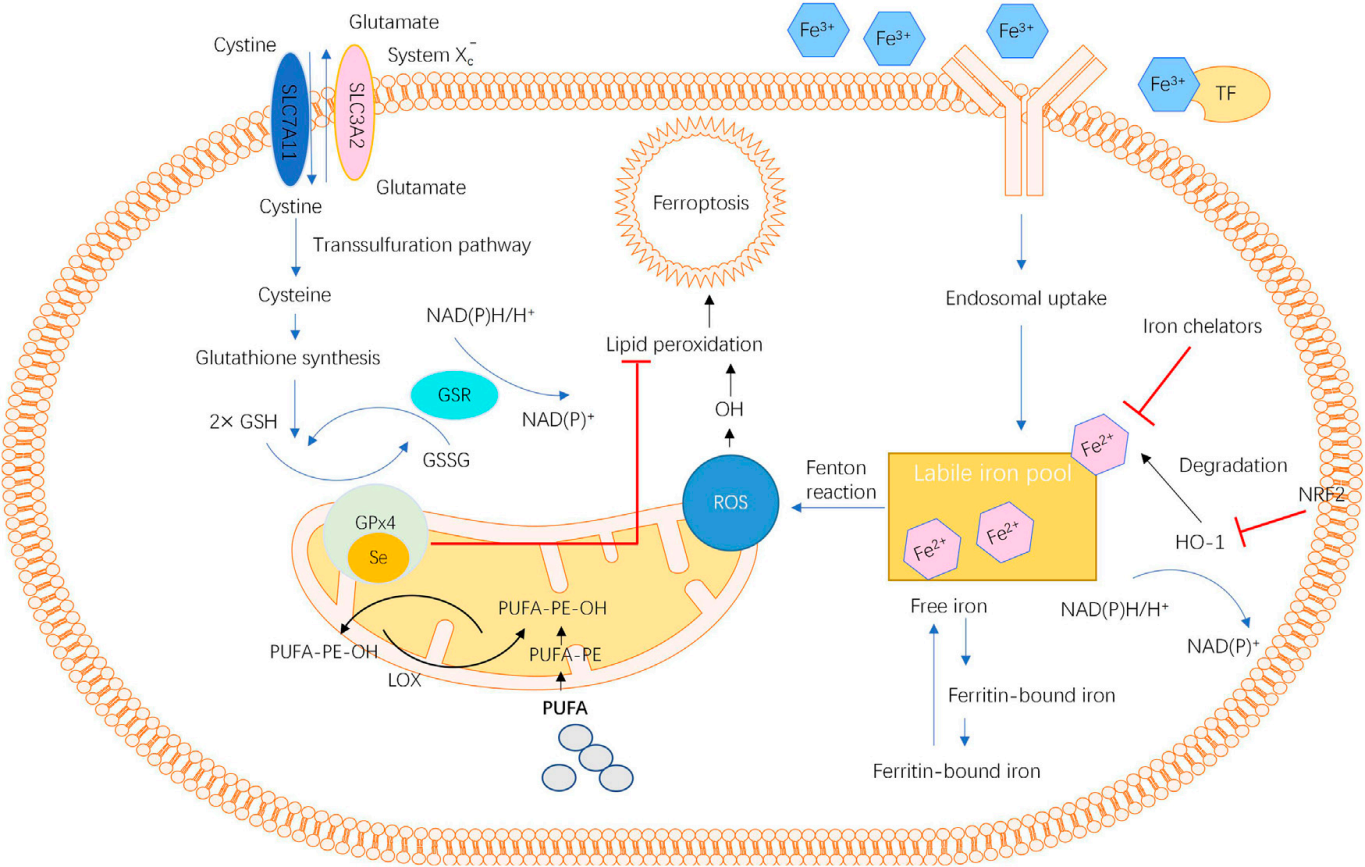
The overall pathways and molecular mechanisms of ferroptosis.

Second, the number of labile iron pools and genes related to iron metabolism also impact the occurrence of ferroptosis (Jiang et al., 2021). Normally, the intracellular iron level is maintained by a precise regulatory system. Under some pathological conditions, such as disturbance in the iron mechanism, repeated bleeding and chronic inflammation with continuous parenchymal cell death, iron is overloaded at the corresponding position (Jiang et al., 2021). Once excessive Fe^2+^ is present in the labile iron pool, oxidative stress is induced by lipid peroxidation via the Fenton reaction catalysed by iron, resulting in a high sensitivity of cells to ferroptosis (Cao and Dixon, 2016; Zhu et al., 2020). In addition to iron, genes regulating iron metabolic processes, such as haem oxygenase 1 (HO-1), also mediate the occurrence of ferroptosis (Cao and Dixon, 2016; Liu J. et al., 2021). Another mediator, mitochondrial ferritin (FtMt), an iron-storage protein located in the mitochondria that plays a significant role in modulating cellular iron metabolism, has been demonstrated to protect cells from erastin-induced ferroptosis (Wang P. et al., 2021; Wang X. et al., 2021; Wang et al., 2016). The overall view of ferroptosis is described in Figure 1.

Emerging evidence shows that ferroptosis is involved in various human diseases, particularly liver diseases (Capelletti et al., 2020; Mao et al., 2020; Yu and Wang, 2021). An important function of the liver is the regulation of iron homeostasis. The liver orchestrates iron transport and storage through gene regulation and maintains the hepatic iron concentration via iron mobilization. Disorder of liver function leads to an imbalance in iron homeostasis, which leads to a series of iron-related diseases, such as anaemia and iron overload (Park et al., 2019; Yu et al., 2020). The characteristics of ferroptosis, such as iron metabolic disorder, amino acid antioxidant system imbalance and lipid peroxide accumulation, are found in different stages of liver disease (Capelletti et al., 2020). Targeting ferroptosis may prevent the pathophysiological development of many liver diseases. The underlying mechanisms include an interplay between antiferroptotic action and other bioactivities, including antioxidative and anti-inflammatory action and the regulation of the immunogenic response (Capelletti et al., 2020; Wu et al., 2021). Therefore, in this study, we summarize the current knowledge regarding the role of ferroptosis in a wide spectrum of liver diseases, including alcoholic liver disease (ALD), nonalcoholic fatty liver disease (NAFLD), hepatitis, fibrosis and hepatocellular carcinoma (HCC), and review agents targeting ferroptosis for liver diseases based on the most up-to-date evidence to provide new therapeutic strategies for liver diseases.

## The Role of Ferroptosis in Liver Diseases

**FIGURE 2:**
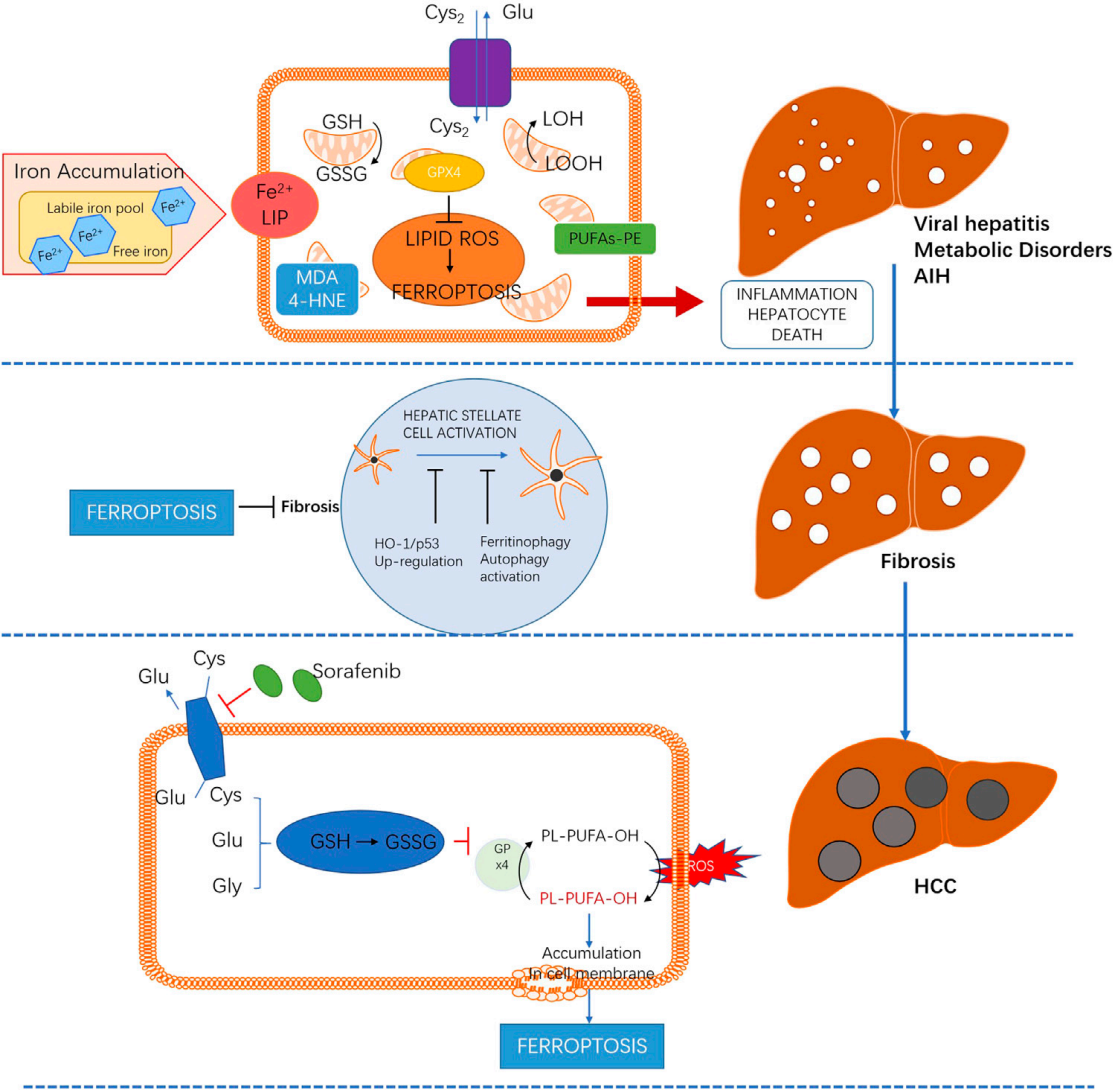
The role and potential mechanism of ferroptosis in different liver diseases.

As a new atypical cell death, ferroptosis plays an important role in the occurrence and development of various liver diseases. Targeting ferroptosis may prevent the pathophysiological progression of several liver injuries, such as liver injury induced by ethanol, NAFLD and immune-mediated hepatitis, which are mediated by lipid peroxidation, inflammatory infiltration and immunogenicity. Meanwhile, promoting ferroptosis can be beneficial for the prognosis of liver fibrosis and HCC. The emerging role of ferroptosis in a wide spectrum of liver diseases is summarized in Figure 2.

### Alcoholic Liver Disease

ALD is a complex process with a wide spectrum of hepatic lesions, from steatosis to cirrhosis, induced by excessive alcohol consumption. During the development and progression of ALD, a large amount of Fe^2+^ accumulates in the liver. In patients with ALD, the level of serum ferritin was found to decrease, while the expression of divalent metal ion transporters was increased in the intestine, resulting in an increase in the serum iron and ferritin levels (Suzuki et al., 2002; Rouault, 2003). NADPH, which acts as a coenzyme of GSH reductase, was found to be dramatically reduced in the livers of alcohol-treated mice (Gao et al., 2017). Moreover, Ptgs2 mRNA, a downstream marker of lipid metabolism during GPX4-regulated ferroptosis, was also upregulated. Another vital part of system X_C_^−^, SLC7A11, which is responsible for maintaining redox homeostasis by importing cystine, was inhibited in the alcoholic liver. These mediators, decreased NADPH, upregulated Ptgs2 and inhibited SLC7A11 and GPX4 facilitated ferroptosis in mice with alcoholic liver injury (Liu C.-Y. et al., 2020).

Studies have shown that ferroptosis inhibitors can significantly alleviate hepatocyte death induced by alcohol (Gao et al., 2017; Liu C.-Y. et al., 2020; Liu J. et al., 2020; Miyata and Nagy, 2020). For instance, ferrostatin-1, a selective inhibitor of ferroptosis, has been demonstrated to reduce alcoholic liver injury in mice (Suzuki et al., 2002). Therefore, targeting ferroptosis might serve as a hepatoprotective strategy for ALD treatment. In a study by Zhou et al. (2019), ferroptosis was identified as a mechanism mediating the detrimental effects of adipose-specific lipin-1 overexpression in mice with alcoholic hepatitis. The overexpression of lipin-1 promoted the synthesis of triacylglycerol and the oxidation of fatty acids and accelerated iron accumulation, leading to the occurrence of ferroptosis and liver injury. Zhou et al. (2020) further reported the protective effects of intestinal sirtuin1 (SIRT1) deficiency by mitigating ferroptosis in mice with alcoholic liver injury. Compared with the wild-type group, ameliorated dysfunctional iron metabolism, increased hepatic glutathione contents, attenuated lipid peroxidation, and downregulated expression of several genes involved in the ferroptosis process were observed in the livers of ethanol-fed mice with intestinal SIRT1 knockout. In another study, however, the liver-specific deletion of SIRT1 in mice disrupted lipin-1 signalling and aggravated alcoholic steatosis (Yin et al., 2014), illustrating the different roles of SIRT1 located in specific sites in response to alcohol exposure.

### Nonalcoholic Fatty Liver Disease

The global incidence rate of NAFLD has been continuously increasing (Wegrzyniak et al., 2021). NAFLD represents a wide range of liver diseases induced by metabolic stress, from simple fatty liver to liver cirrhosis. Nonalcoholic steatohepatitis (NASH) is an intermediate stage of NAFLD. In the pathogenesis of NAFLD, oxidative stress caused by lipid peroxide accumulation is considered an important initial factor, and iron deposition caused by metabolic disorder also serves as an aggravating factor of NASH by increasing the risk of hepatocyte expansion, inflammation and fibrosis (Yang et al., 2020; Gao et al., 2021; Ota, 2021). The administration of a high-iron diet aggravated oxidative stress and the inflammatory response, accelerating the progression of NAFLD to NASH in a mouse model (Videla and Valenzuela, 2021). Thus, ferroptosis has been intensively demonstrated to accelerate the development of hepatic lesions in NAFLD (Tsurusaki et al., 2019; Magusto et al., 2020). A study reported that ferroptosis occurred in the earliest stages of NASH and that targeting ferroptosis inhibition could effectively protect hepatic cells from necrotic death (Tsurusaki et al., 2019). Meanwhile, it has been shown that inhibiting subsequent immune cell infiltration, the inflammatory response and lipid accumulation did not have an effect on alleviating the death of liver cells (Tsurusaki et al., 2019), indicating that ferroptosis might be a special therapeutic target against the onset of NASH.

Several studies attempted to target ferroptosis to alleviate the progression of NAFLD in a mouse model (Tsurusaki et al., 2019; Chen et al., 2020; Mao et al., 2020; Gao et al., 2021; Jia et al., 2021; Wu et al., 2021). Inhibitors of ferroptosis, such as liproxstatin-1, repress hepatic lipid peroxidation and its associated cell death, resulting in a decreased severity of NASH (Tsurusaki et al., 2019; Li X. et al., 2020; Qi et al., 2020). In a previous study, the ferroptosis inducer RSL3 aggravated hepatic steatosis and inflammation in methionine/choline-deficient (MCD) diet-induced NASH mice, while the treatment with a GPX4 activator, sodium selenite, relieved the RSL3-induced lipid peroxidation and cell death, thereby reducing the severity of NASH (Qi et al., 2020). The upregulation of enolase 3 (ENO3), which encodes the β-subunit of enolase, aggravated the progression of NASH by inducing ferroptosis by increasing GPX4 expression in a NASH mouse model (Lu et al., 2021), suggesting that the inhibition of ENOS might be beneficial for NASH treatment.

### Viral Hepatitis

Hepatitis C virus (HCV) can induce oxidative stress and inhibit liver ferritin, resulting in the upregulation of duodenal membrane iron transporter-1 (Zou and Sun, 2017). Meanwhile, studies have revealed that transferrin receptor 1 (TfR1) can cause changes in iron metabolism in hepatocytes infected with HCV, facilitating the replication of viral genetic material and the translation of transcripts (Martin and Uprichard, 2013). This suggests that HCV can cause hepatocytes intracellular iron content of HCV patients which was closely related to the severity of the disease. Additionally, the levels of serum transferrin, cellular iron and ferritin were significantly increased in HCV patients even after 24 h of treatment with pegylated interferon combined with ribavirin (Martin and Uprichard, 2013). In a recent study, however, it was demonstrated that the cellular sensitivity to ferroptosis and permissiveness for HCV replication were dictated by fatty acid desaturase 2 (FADS2) (Yamane et al., 2021). The inhibition of FADS2 significantly enhanced HCV replication, whereas erastin, a ferroptosis-inducing compound, reduced the conformation of the HCV replicase and sensitized it to antiviral drugs targeting the viral protease. Thus, FADS2, which mediates the noncanonical desaturation of oleate to Mead acid and other highly unsaturated fatty acids, is considered a rate-limiting factor of ferroptosis. Pharmaceuticals regulating the ferroptosis pathway might attenuate the replication of HCV. In another recent *in vitro* study (Komissarov et al., 2021), it was found that in human cells infected with hepatitis A virus 3C protease (3Cpro), cell death induced by 3Cpro was accompanied by intense lipid peroxidation and efficiently suppressed by ferroptosis inhibitors. Although this study was not enough to confirm the biological role of 3Cpro as a ferroptosis inducer, a relationship between the ability of 3Cpro to induce ferroptosis and the viral life cycle was suggested. These studies extend our knowledge regarding the mechanism and role of ferroptosis in viral hepatitis. In a recent study, HBV X protein (HBx), which is an essential HBV regulatory protein involved in the development of HBV-associated severe liver disease, was found to facilitate ferroptosis in acute liver failure via EZH2-mediated SLC7A11 suppression (Liu G.-Z. et al., 2021).

### Autoimmune Hepatitis

Autoimmune liver disease represents a group of chronic liver diseases caused by immune-mediated injury and is associated with a significant risk of developing end-stage liver disease if not treated in a timely manner with effective therapy (Sahebjam and Vierling, 2015; Sucher et al., 2019). However, the pathological mechanism of autoimmune hepatitis (AIH), particularly the detailed mechanism of hepatocyte death, has not been fully clarified. In a caveolin-1 (Cav-1)-deficient mouse model with ConA triggering, the knockout of Cav-1 significantly inducing ferroptosis and more serious AIH by promoting the massive accumulation of ROS and reactive nitrogen species induced by ConA (Deng et al., 2020). Fer-1, a known ferroptosis inhibitor, can reverse ferroptosis and attenuate liver injury induced by ConA. Intervention with ferroptosis inhibitors like Fer-1 and Indoleamine 2,3-dioxygenase 1 (IDO1), a type of intracellular haem enzyme and immune regulator which is associated with the production of Fe^2+^, could mitigate ferroptotic cell death and nitrative stress in mice treated with ConA (Zeng et al., 2020). The mechanism underlying ferroptosis of IDO1 is attributed to its role as a system XC modulator and excessive nitrative stress. All of these indicated that the effect of ferroptosis on immune-mediated hepatitis, indiating the potential value of targeting ferroptosis to improve the treatment of AIH (Shojaie et al., 2020).

### Fibrosis

Hepatic fibrosis is a type of disease due to the wound-healing response of the liver to various causes of chronic injury. The main pathological features of hepatic fibrosis are the activation of hepatic stellate cells (HSCs) and excessive deposition of extracellular matrix (ECM) in the liver (Smid, 2020; Sun et al., 2020). The abnormal proliferation of a large amount of fibrous connective tissue in the portal area destroys the normal structure and physiological function of the liver (Ramos-Tovar and Muriel, 2020; Smid, 2020). Therefore, inhibiting the activation and proliferation of HSCs is an effective therapeutic strategy for liver fibrosis. In recent years, the emerging role of ferroptosis in the development of liver fibrosis has attracted increasing attention. Compared with the normal liver, significantly increased iron ions in HSCs and lipid peroxidation were observed in the fibrotic liver, suggesting that ferroptosis might be involved in the process of liver fibrosis (Mehta et al., 2019; Yu et al., 2020). Targeting ferroptosis in HSCs has become a promising therapeutic approach for liver treatment.

Various genes and proteins have been identified as modulators of ferroptosis in HSCs. HO-1 is a gatekeeper in response to various insults in multiple pathological conditions by mediating ferroptosis. In a rat liver fibrosis model, the upregulation of HO-1 induced by magnesium isoglycyrrhizinate promoted the accumulation of iron and lipid peroxide, resulting in the ferroptosis of HSCs, while the antifibrotic effect of magnesium isoglycyrrhizinate was eliminated when ferrostatin-1 was used or HO-1 was silenced (Sui et al., 2018). The RNA binding proteins zfp36 ring finger protein 36 (zfp36/TTP) and elav-like RNA binding protein 1 (elavl1/HuR) also play an important role in regulating ferroptosis in HSCs (Zhang Z. et al., 2018; Zhang Z. et al., 2020a). Studies have demonstrated that the downregulation of zfp36 via abrogating beclin mRNA stability and the upregulation of elavl1 by reversing ATG16L1 mRNA decay mediated by sorafenib/erastin could promote ferroptosis in HSCs to reduce liver fibrosis in mice (Zhang Z. et al., 2020a; Shen et al., 2021). The binding of zfp36/elavl1 to downstream target genes regulates ferritin autophagy in HSCs and then releases iron ions, leading to a large amount of ROS through the Fenton reaction and HSC ferroptosis.

Studies have indicated that the bromine-containing protein 7 (BRD7)-p53 solute carrier family 25 members 28 (BRD7-P53-SLC25A28) axis plays a critical role in regulating HSC ferroptosis as evidenced by knockout and overexpression function assays (Zhang Z. et al., 2020b). The increased expression of BRD7 could promote p53 mitochondrial translocation by directly binding the p53 N-terminal transactivation domain, which interacts with downstream SLC25A28 to form a complex to induce ferroptosis. In addition, the upregulation of p53 expression mediated by artemether suppressed recombinant protein of solute carrier family 7 member 11 (SLC7A11), indirectly leading to the inactivation of GPX4 and finally facilitating ferroptosis in HSCs and improving fibrotic liver. Liver transferrin (TRF), a serum-abundant metal-binding protein, has been shown to alleviate liver fibrosis via ferroptosis (Yu et al., 2020). It has been shown that the knockout of TRF in hepatocytes from mice resulted in the accumulation of non-transferrin bound iron in the liver, which further aggravated liver fibrosis mediated by a high iron diet, while the specific knockout of TRF and solute carrier family 39 member 14 (SLC39A14) could significantly reduce the accumulation of iron in the liver, leading to improved liver fibrosis mediated by a high iron diet or carbon tetrachloride injection (Jenkitkasemwong et al., 2015). These studies suggest that liver transferrin and BRD7 play a protective role in maintaining liver function and provide a potential therapeutic target for preventing liver fibrosis induced by iron overload.

### Hepatocellular Carcinoma

HCC is the most common type of liver cancer and represents approximately 90% of all cases with a low survival rate (Llovet et al., 2021). To date, several regulatory pathways have been identified to mediate the ferroptoic response in liver tumour cells. The main regulated mediators are system X_C_^−^ and GPX4 (Zhang X. et al., 2020). MicroRNA-214-3p inhibited GSH synthesis and induced ferroptosis in liver cancer cells by inhibiting the expression of agonist transcription factor 4 (Bai et al., 2020). Another critical mediator, p53, a tumour suppressor gene, downregulated SLC7A11 transcription, affecting the activity of system X_C_^−^, followed by ferroptosis in liver cancer cells (Ou et al., 2016). The other pathway involved in p53-mediated ferroptosis is related to spermine/spermidine-n1-acetyl. The upregulated expression of spermine/spermidine-n1-acetyltransferase 1 mediated by p53 increased the level of arachidonic acid 15 lipoxygenase and promoted the accumulation of cytoplasmic peroxides, which finally induced ferroptosis (Zhang et al., 2019). Another tumour suppressor gene, BRCA1-related protein 1, was found to promote ferroptosis by suppressing SLC7A11 (Zhang Y. et al., 2018). An iron-containing outer mitochondrial membrane protein, CDGSH iron sulfur domain 1 (CISD1), negatively regulates ferroptotic HepG2 death by protecting against mitochondrial lipid peroxidation (Yuan et al., 2016).

Sorafenib is a key licensed first-line therapy for advanced HCC that could enhance patient survival, although drug resistance to sorafenib limits its usefulness to patients (Zhu et al., 2017). Given that sorafenib is the only anticancer agent that can cause ferroptosis in liver cancer patients, the function of ferroptosis in sorafenib resistance has received a lot of attention (Lachaier et al., 2014; Tang et al., 2019). However, promoting ferroptosis in liver cancer cells, suppressing the expression of metallothionein 1G (MT1G) and oxidative stress-related protein sigma 1 receptor (S1R) can both improve the drug resistance of liver cancer to sorafenib (Sun, et al., 2016a). The retinoblastoma (RB) protein belongs to a family of protein that regulates gene transcription in eukaryotic cells. The major function of RB is to act as a negative regulator of cell proliferation and cell cycle activities. The loss of RB protein function is very common in HCC. It has been observed that when liver cancer cells lacking the RB protein are exposed to sorafenib, their cell mortality is 2∼3 times higher than that of liver cancer cells with normal RB protein levels, suggesting that evaluating the RB status could be used to judge the drug resistance of HCC patients to sorafenib (Louandre et al., 2015).

Ceruloplasmin (CP) has been discovered to suppress ferroptosis in HCC cells via controlling iron homeostasis (Shang et al., 2020). The suppression of CP significantly increased the accumulation of Fe^2+^ and ROS, facilitating ferroptosis in HCC cells induced by erastin, a ferroptosis inducer. As a result of downregulated peroxidase gene expression, erastin could significantly inhibit the expression of GA binding protein transcription factor subunit β1 (GABPB1) in HCC cells, resulting in the continuous accumulation of intracellular ROS and malondialdehyde and leading to ferroptosis in cancer cells (Qi et al., 2019; Zhang et al., 2019; Tang D. et al., 2021). Hence, combination therapy for HCC patients may solve some drug resistance problems and improve the clinical effect of sorafenib.

In addition, the expression of long-chain acyl CoA synthase long chain family member 3 (ACSL3) and ACSL4 was dramatically elevated in hepatoma cells, and ACSL4 was involved in erastin-induced ferroptosis via 5-hydroxyeicosatetraenoic acid-mediated lipotoxicity (Ndiaye et al., 2020). Bai et al. found that using sorafenib in combination with a sigma receptor antagonist could facilitate ferroptosis in liver cancer cells (Bai et al., 2019; Bai et al., 2017). Due to Nrf2 inactivation and subsequent ROS accumulation and antagonized ferroptosis by reducing GSH consumption, the sigma receptor was passively upregulated in hepatoma cells treated with sorafenib, while haloperidol, a sigma receptor antagonist, reversed this effect (Bai et al., 2017; Bai et al., 2019).

Furthermore, nanoparticle medicines open up new possibilities for inducing ferroptosis in liver cancer cells in situ. Tang et al. (Tang et al., 2019) placed sorafenib with manganese silicon nanoparticles, which caused tumor cells to undergo ferroptosis by rapidly devouring intracellular GSH. Nanoparticles recombined with the low-density lipoprotein docosahexaenoic acid (LDL-DHA) can lead to ferroptosis and a reduction in tumors in rats by promoting lipid peroxidation in liver cancer cells while lowering GSH and GPX4 (Nie et al., 2018; Tang et al., 2019; Shan et al., 2020). Therefore, targeting ferroptosis enhancement in liver cancer cells might be a new strategy to address sorafenib resistance.

## Therapeutic Strategies Targeting Ferroptosis for Liver Diseases

Given the critical role of ferroptosis in liver diseases mentioned above, we further reviewed currently available studies that used drugs as inducers or inhibitors of ferroptosis to treat liver diseases, including some Chinese medicines, compounds from natural products and Western drugs. The implementation of ferroptosis is closely associated with iron, ROS and PUFAs; thus, numerous genes and signalling pathways related to iron metabolism, ROS production and lipid synthesis have been noted to likely mediate vulnerability to ferroptosis (Capelletti et al., 2020; Mao et al., 2020; Yu and Wang, 2021).

### Compounds Derived From Chinese Medicines and Natural Products

Several compounds derived from Chinese medicines and natural products have been proven to protect the liver from various injuries via ferroptosis regulation, such as ginkgolide B, artemether, artesunate, chrysophanol, piperlongumine and puerarin. Ginkgolide B (GB), a main constituent of *Ginkgo biloba* extracts, was demonstrated to reduce lipid accumulation and ameliorate NAFLD in obese mice in association with ferroptosis regulation (Yang et al., 2020). Both HFD-fed mice and palmitic acid and oleic acid (PA/OA)-induced HepG2 cells showed a ferroptosis-based panel of biomarkers, such as excessive iron with increased transferrin receptor-1 (TFR1), reduced ferritin heavy chain-1 (FTH1) and inhibited Nrf2 activity, which further induced the GPX4 and HO-1 levels (Yang et al., 2020). GB treatment exerted an effect on ferroptosis by reducing lipid accumulation and oxidative stress via the possible mechanism of Nrf2 signalling pathway regulation. When cells are damaged or cancerous, p62 inactivates Keap1 to promote the activation and entry of Nrf2 into the nucleus (Jenkins and Gouge, 2021). Then, Nrf2 forms a dimer with fibrosarcoma protein and binds antioxidant response elements (AREs) to regulate downstream quinone oxidoreductase 1 (NQO1), HO-1, ferritin heavy chain 1 (fth1) and other genes related to ferroptosis (Sun et al., 2016b). Furthermore, Nrf2 can promote the expression of metallothionein 1G (MT1G), an important negative regulator of ferroptosis, through the cystathionase pathway, leading to the resistance of cancer cells to sorafenib (Sun et al., 2016a). Therefore, Nrf2 may be an important target involved in ferroptosis and future treatment for various liver diseases.

Artemether is a commonly used artemisinin derivative that improves glucose and lipid metabolism by regulating the inflammatory response in db/db mice (Fu et al., 2020). Artemether could upregulate the expression of p53 and suppress SLC7A11, indirectly leading to the inactivation of GPX4 and HSC ferroptosis in the fibrotic liver. Moreover, in addition to targeting p53-SLC7A11, artemether could increase the iron ion levels in HSCs by inhibiting the ubiquitination-mediated degradation of iron regulatory protein 2, thereby generating a large amount of ROS to promote ferroptosis (Li Y. et al., 2020). A recent study also showed that artesunate can reduce liver fibrosis by regulating ferritin autophagy-mediated ferroptosis in HSCs, while lysosome-specific inhibitors can reverse this effect (Kong et al., 2019). Recently, it was also found that chrysophanol promoted HSC ferroptosis mediated by hepatitis B virus X protein by increasing the ROS levels and reducing SLC7A11 expression (Kuo et al., 2020). In addition, piperlongumine from *Piper longum* and puerarin can affect the occurrence of ferroptosis by regulating the level of ROS (Xu et al., 2018).

Notably, some natural products and compounds have been proven to target lipid peroxidation to inhibit ferroptosis, such as brown rice extract, baicalin, and vitamin E (Xu et al., 2018). Brown rice extract was observed to inhibit ferroptosis and, thus, improve the lipid peroxidation and cytotoxicity caused by GPX4 inactivation (Sakai et al., 2017). Baicalin inhibited ferroptosis by inhibiting lipid peroxidation and reducing free iron accumulation, and its inhibitory effect was even better than that of some typical ferroptosis inhibitors (Xie et al., 2016). Other inhibitors, such as vitamin E, butylated hydroxytoluene and tert-butylhydroxyanisole, were also noted to suppress ferroptosis by preventing lipid peroxidation (Kim et al., 2020; Krummel et al., 2021). Although the mechanism of many traditional Chinese medicines and their active components in ferroptosis are not fully understood, increasing evidence indicates that some natural compounds might exert hepatoprotective effects by regulating ferroptosis, and further studies in this field are expected.

### Western Medicines Such as Sorafenib and Some Nanoparticle Drugs

Numerous Western medicines have been noted to mediate ferroptosis by targeting GSH. Cysteine sulfonimide promoted lipid peroxidation by inhibiting the rate-limiting enzyme of GSH synthesis, glutamate cysteine ligase (GCL), reducing the GSH levels and GPX4 activity, and then, inducing ferroptosis in various cancer cells (Yang et al., 2014). Cisplatin can directly combine with GSH to form a Pt-GSH complex, resulting in the inactivation of GSH and GPX4, thereby promoting ferroptosis (Yang et al., 2014; Guo et al., 2018; Nishizawa et al., 2018). Furthermore, a significant synergistic antitumour effect was observed when cisplatin and erastin were jointly applied to human lung cancer cells and colon cancer cells. In addition, the synthetic compound diphenyleneiodonium chloride 2 has a similar drug action mechanism and induces ferroptosis by consuming GSH in cells (Guo et al., 2018). Other drugs, such as buthinonine sulfoximine (BSO) and sulfasalazine, were also found to impact system X_C_^−^ and cause GSH depletion to induce ferroptosis (Reliene and Schiestl, 2006; Albano et al., 2015).

Another ferroptosis-related gene targeted by several Western medicines and nanoparticle drugs is ACSL4, which is an important isozyme for PUFA metabolism with an essential role in proferroptotic processes (Cui et al., 2021). Thiazolidinediones, such as rosiglitazone, pioglitazone and troglitazone, can specifically inhibit the expression of ACSL4 to protect cells from ferroptosis and lipid peroxidation induced by RSL3, but the underlying mechanism has not been fully revealed (Doll et al., 2017). Among them, although troglitazone has a low inhibitory effect on ACSL4, it may have inherent antioxidant activity due to its 6-chromogenoalkanol structure, which is the strongest inhibitor of ferroptosis among thiazolidinediones (Doll et al., 2017). Regarding ROS and iron accumulation, several drugs can regulate these factors. In a rat liver fibrosis model, the upregulation of HO-1 induced by magnesium isoglycyrrhizinate promoted the accumulation of iron and lipid peroxide, resulting in the ferroptosis of HSCs, while the antifibrotic effect of magnesium isoglycyrrhizinate was eliminated when ferrostatin-1 was used or HO-1 was silenced (Sui et al., 2018). In addition, according to recent studies, various nanomaterials can coordinate loaded drugs to regulate ferroptosis by affecting the levels of ROS and GSH and the Fenton reaction (Sang et al., 2019; Hsieh et al., 2021; Xiong et al., 2021).

As mentioned above, the discovered ferroptosis inducers mainly induce ferroptosis by targeting system X_C_^−^, GSH, GPX4, iron ions and ROS. In particular, erastin is the first specific ferroptosis inducer that can target the inhibition of system X_C_^−^, affect the synthesis of GSH and promote ferroptosis in various cells (Zhao et al., 2020; Li et al., 2021). However, the characteristics of poor water solubility and unstable metabolism limit its application *in vivo*. Researchers have modified its structure and introduced piperazinyl into erastin to obtain piperazine-erastin, which significantly improved the water solubility and stability, and the effect intensity of inducing ferroptosis was similar to erastin (Yu M. et al., 2019; Zhao et al., 2020; Zheng et al., 2021). Moreover, sulfasalazine and sorafenib, which are widely used in the clinic and have achieved good therapeutic effects, have been approved by the FDA as ferroptosis inducers (Yu H. et al., 2019; Zheng et al., 2021).

In recent years, agents serving as ferroptosis inhibitors have also been highlighted with the underlying mechanism of reducing free iron and ROS, inhibiting lipid production or lipid peroxidase activity, such as ferrostatin-1, liproxstatin-1, butylated hydroxytoluene (BHT), and butylated hydroxyanisole (BHA). Ferrostatin-1 is a specific ferroptosis inhibitor. On the one hand, Ferrostatin-1 protects against ferroptosis induced by erastin or RSL by inhibiting lipid and ROS accumulation (Miotto et al., 2020; Tang W. et al., 2021). On the other hand, Ferrostatin-1 can also downregulate the expression of prostaglandin endoperoxide synthase 2 and upregulate the expression of GPX4 and Nrf2 proteins, reduce the level of ROS and lipid peroxidation, and protect cells from oxidative toxicity and ferroptosis (Miotto et al., 2020; Chen et al., 2021; Xu et al., 2021). BHT and BHA protect liver cells from injury via ferroptosis inhibition mainly by preventing lipid peroxidation. Therefore, it is of great significance to explore the mechanism of ferroptosis inducers and inhibitors and develop new targeted ferroptosis drugs for the treatment of inflammatory liver diseases or cancerous liver.

## Conclusion and Perspectives

Based on the special biological characteristics of ferroptosis and in-depth research investigating different treatment approaches for liver diseases, this newly discovered mode of cell death has attracted increasing attention. Targeting the inhibition of ferroptosis plays an important role in protecting against various liver injuries mediated by lipid peroxidation, inflammatory infiltration and immunogenicity. In contrast, promoting ferroptosis can be beneficial for impeding the prognosis of liver fibrosis and HCC diseases. In particular, drug resistance is a critical problem in the treatment of liver tumours; thus, finding a novel cell death mechanism for drug resistance is a new breakthrough in tumour treatment. Sorafenib can increase the sensitivity of cancer cells to ferroptosis by changing the redox of drug-resistant genes in the study of drug resistance in liver cancer, which can provide a new idea for the study of drug resistance in liver cancer. Although studies investigating the molecular mechanism and signalling pathway of ferroptosis in the process of liver diseases are increasing, there are still many concerns that need to be further addressed. For example, the exact molecular events involved in the final cell death caused by ferroptosis are not completely unravelled. Currently, it is generally believed that phospholipid peroxide damages the integrity of the membrane. However, a recent study found that phospholipids containing two PUFA tails are important factors driving ferroptosis, suggesting that lipid crosslinking may be an aspect of membrane damage during ferroptosis. In this case, it may be that cross-linked lipids limit the fluidity of membrane components, resulting in the failure of some important membrane-related functions and cell death. Of course, there are other possibilities, such as the decomposition of oxidized PUFA-PLs into reactive electrophiles and then destruction of other macromolecules. Notably, most existing reports in the literature focusing on the role of ferroptosis in liver diseases are indirect studies; thus, it is difficult to determine the direct action. However, therapeutic effects cannot be achieved if only antioxidants are used to inhibit lipid peroxidation. Therefore, understanding its specific mechanism and causality is critical for analysing the role of ferroptosis in liver diseases. Moreover, the precise *in vivo* specific markers of ferroptosis remain unclear. The iron ion and lipid peroxidation levels and the increased expression of transferrin receptors are currently considered potential markers of ferroptosis. Therefore, with the deepening of research, the determination of accurate *in vivo* biomarkers of ferroptosis in the future is of great importance for understanding the physiological function and therapeutic potential of this cell death mode and can provide an important basis for the clinical diagnosis and treatment of diseases. Finally, a clinical study investigating whether targeting ferroptosis can achieve benefits in the treatment of liver diseases has not been reported. Clinical trials using specific ferroptosis inducers or inhibitors in various liver diseases are expected. In conclusion, an improved understanding of the role of ferroptosis could provide a new therapeutic strategy for the treatment of liver diseases and place ferroptosis under the spotlight of future translational medicine.
